# Trastuzumab mediates antibody-dependent cell-mediated cytotoxicity and phagocytosis to the same extent in both adjuvant and metastatic HER2/neu breast cancer patients

**DOI:** 10.1186/1479-5876-11-307

**Published:** 2013-12-12

**Authors:** Branka Petricevic, Johannes Laengle, Josef Singer, Monika Sachet, Judit Fazekas, Guenther Steger, Rupert Bartsch, Erika Jensen-Jarolim, Michael Bergmann

**Affiliations:** 1 Department of Surgery, Medical University of Vienna, Vienna General Hospital, Waehringer Guertel 18-20, Vienna A-1090, Austria; 2 Comparative Medicine, Messerli Research Institute of the University of Veterinary Medicine Vienna, Medical University of Vienna and University of Vienna, Veterinaerplatz 1, Vienna A-1210, Austria; 3 Comparative Immunology and Oncology, Department of Pathophysiology and Allergy Research, Center of Pathophysiology, Infectiology and Immunology, Medical University of Vienna, Vienna General Hospital, Waehringer Guertel 18-20, Vienna A-1090, Austria; 4 Department of Oncology, Medical University of Vienna, Vienna General Hospital, Waehringer Guertel 18-20, Vienna A-1090, Austria; 5 Comprehensive Cancer Center Vienna, Spitalgasse 23, Vienna A-1090, Austria

**Keywords:** ADCC, ADCP, HER2/neu, Breast cancer, Trastuzumab

## Abstract

**Background:**

Monoclonal antibodies (mAb), such as trastuzumab are a valuable addition to breast cancer therapy. Data obtained from neoadjuvant settings revealed that antibody-dependent cell-mediated cytotoxicity (ADCC) is a major mechanism of action for the mAb trastuzumab. Conflicting results still call into question whether disease progression, prolonged treatment or concomitant chemotherapy influences ADCC and related immunological phenomena.

**Methods:**

We analyzed the activity of ADCC and antibody-dependent cell-mediated phagocytosis (ADCP) of peripheral blood mononuclear cells (PBMCs) from human epidermal growth factor receptor 2 (HER2/neu) positive breast cancer patients receiving trastuzumab therapy either in an adjuvant (n = 13) or metastatic (n = 15) setting as well as from trastuzumab treatment-naive (t-naive) HER2/neu negative patients (n = 15). PBMCs from healthy volunteers (n = 24) were used as controls. ADCC and ADCP activity was correlated with the expression of antibody binding Fc-gamma receptor (FcγR)I (CD64), FcγRII (CD32) and FcγRIII (CD16) on CD14+ (monocytes) and CD56+ (NK) cells, as well as the expression of CD107a+ (LAMP-1) on CD56+ cells and the total amount of CD4+CD25+FOXP3+ (T_reg_) cells. In metastatic patients, markers were correlated with progression-free survival (PFS).

**Results:**

ADCC activity was significantly down regulated in metastatic, adjuvant and t-naive patient cohorts as compared to healthy controls. Reduced ADCC activity was inversely correlated with the expression of CD107a on CD56+ cells in adjuvant patients. ADCC and ADCP activity of the patient cohorts were similar, regardless of treatment duration or additional chemotherapy. PFS in metastatic patients inversely correlated with the number of peripheral T_reg_ cells.

**Conclusion:**

The reduction of ADCC in patients as compared to healthy controls calls for adjuvant strategies, such as immune-enhancing agents, to improve the activity of trastuzumab. However, efficacy of trastuzumab-specific ADCC and ADCP appears not to be affected by treatment duration, disease progression or concomitant chemotherapy. This finding supports the application of trastuzumab at any stage of the disease.

## Background

Immunotherapy based on monoclonal antibodies (mAb) targeting tumor-associated antigens (TAA) such as trastuzumab (anti-human epidermal growth factor receptor 2; HER2/neu) is clinically effective [1-6] and has thus become the standard of care for women with HER2/neu expressing breast cancer [7-9]. However, when administered as a monotherapy, this drug only yields response rates of 12% to 35% [10-12]. Understanding the key mechanisms, which underlie the therapeutic efficacy of trastuzumab would enable oncologists to exclude patients from therapy who probably do not benefit, and might also help to assist in the development of adjuvant therapies that enhance the therapeutic effects of this antibody.

It has been postulated that trastuzumabs’ anti-tumor activity depends on multiple direct or indirect cytostatic and/or cytotoxic effects [13-17]. Direct binding can induce HER2/neu down regulation and the alteration of several HER2/neu dependent cellular signaling pathways leading to tumor growth inhibition. However, loss of HER2/neu expression [18,19] and cell signal transduction inhibition was not necessarily relevant for the development of resistance to trastuzumab *in vivo* and in a nude mouse model [20,21].

Studies on animal models revealed that the therapeutic activity of trastuzumab critically depends on the involvement of Fc-gamma receptor (FcγR)-expressing lymphocytes [22,23]. With respect to FcγRI (CD64) and FcγRIII (CD16) it could be demonstrated that mice lacking those two receptors were unable to mount protective immune responses against a virus-encoded tumor-specific antigen [24]. These studies indicate that antibody-dependent cell-mediated cytotoxicity (ADCC) is a major mechanism of action for mAb. Moreover, trastuzumab present in breast cancer patients’ serum after neoadjuvant application significantly enhanced their ADCC activity [25,26]. It is important to note that ADCC correlated with therapeutic response in that limited number of patients. In a metastatic setting the correlation of ADCC and therapeutic success is less clear. Some authors found that higher ADCC was predictive of the lack of disease progression [27], while other pilot studies did not observe a significant association [28,29]. Due to contradictory results, ADCC is currently regarded to be insufficient for the treatment of metastatic cancer [30]. This would imply that different mechanisms of trastuzumab, which are able to induce cell death, are relevant at the later stages of the disease. Thus, we concluded that more clinical data is required to gain better understanding if disease progression and prolonged treatment affects ADCC and its related immune parameters, which could then allow a functional design for immune enhancing strategies and their proper applications.

Therefore, we investigated the impact of disease status, adjuvant or metastatic, on ADCC and antibody-dependent cell-mediated phagocytosis (ADCP) in HER2/neu breast cancer patients receiving trastuzumab. We also included trastuzumab treatment-naive (t-naive) patients, which were HER2/neu negative. Recently, a three-color flow cytometric method has been developed to evaluate simultaneously ADCC and ADCP [31]. This technique has been further adapted for trastuzumab and HER2/neu overexpressing cancer cells [32]. Using this method, we analyzed the reactivity of peripheral blood mononuclear cells (PBMCs) of adjuvant, metastatic and t-naive patients. Further, we correlated this reactivity with the expression of Fc-gamma receptors and the amount of regulatory T (T_reg_) cells as a surrogate parameter for tumor-associated immunosuppression.

## Materials and methods

### Study collective

15 metastatic and 13 adjuvant HER2/neu breast cancer patients were enrolled in the study. All received trastuzumab, starting with a loading dose of 8 mg/kg, followed by 6 mg/kg in a standard 3 week cycle. 15 HER2/neu negative breast cancer patients served as a trastuzumab treatment-naive (t-naive) group (patient characteristics are shown in Table 1). Adjuvant patients received trastuzumab treatment for 1 year, whereas metastatic patients were treated with trastuzumab until intolerable toxicities, disease progression and switch to lapatinib or death occurred. Blood samples were taken prior to a new trastuzumab application. Patients did neither receive radiotherapy nor underwent any surgical intervention for at least 4 weeks prior to blood sampling. 24 healthy volunteers served as a control group for the patient collective. Clinical responses were determined according to response evaluation criteria in solid tumors (RECIST). Progression-free survival (PFS) was assessed in a 1-year follow-up.

**Table 1:** Clinical characteristics of participants

	**Healthy**	**Adjuvant**	**Metastatic**	**T-Naive**
Number, N	28	13	15	15
Age, years				
Median	30	58	68	64
Range	23-55	37-82	40-75	34-74
HER2/neu receptor, N (%)				
Positive	N.A.	13 (100%)	15 (100%)	0 (0%)
Negative		0 (0%)	0 (0%)	15 (100%)
Estrogen receptor, N (%)				
Positive	N.A.	9 (69%)	8 (44%)	11 (70%)
Negative		4 (31%)	7 (46%)	4 (30%)
Progesterone receptor, N (%)				
Positive	N.A.	5 (38%)	1 (10%)	7 (40%)
Negative		8 (62%)	14 (90%)	8 (60%)
Trastuzumab, N	N.A.	13 (100%)	15 (100%)	N.A.
Median application time, months		7	58	
IQR		5	45	
Chemotherapy, N (%)				
FEC	N.A.	1 (10%)		1 (10%)
FEC-Doc				1 (10%)
EC				3 (20%)
EC-D				2 (10%)
CMF		1 (10%)		
Docetaxel + Epirubicin		3 (20%)	1 (10%)	
Docetaxel		1 (10%)		
Vinorelbin		1 (10%)	1 (10%)	
Carboplatin			1 (10%)	
Paclitaxel				1 (10%)

N: number of individuals; N.A.: not applicable; IQR: Interquartile range; FEC: 5-Fluorouracil, Epirubicin and Cyclophosphamide; FEC-Doc: 5-Fluorouracil, Epirubicin, Cyclophosphamide and Docetaxel; EC: Epirubicin and Cyclophosphamide; EC-D: Epirubicin, Cyclophosphamide and Docetaxel; CMF: Cyclophosphamide, Methotrexate and 5-Fluorouracil.

### Cell line

The human breast adenocarcinoma cell line SKBR3 (ATCC, Manasses, VA, USA) was grown in RPMI 1640 medium with 2 mM stable l-glutamine (PAA Laboratories, Pasching, Austria) and an addition of 10% heat inactivated fetal calf serum (FCS; Linaris, Wertheim-Bettingen, Germany). SKBR3 cells served as target (T) cells in the ADCC/ADCP assay.

### Isolation of PBMCs

Peripheral blood was drawn into ethylene diamine tetra-acetic acid (EDTA) tubes (Greiner Bio-One, Kremsmünster, Austria) and processed at room temperature (RT). PBMCs were isolated by Ficoll-Paque (Amersham Pharmacia Biotech, Uppsala, Sweden) gradient centrifugation. One aliquot of the PBMCs was resuspended in RPMI 1640 medium with 2 mM stable glutamine (PAA Laboratories) containing 25% FCS (Linaris) and 5% dimethyl sulfoxide (DMSO; Sigma Aldrich, Steinheim, Germany) followed by an intermediate cooling over night at -80°C, and stored afterwards in liquid nitrogen for later marker analysis. he rest of the freshly isolated PBMCs was used as effector (E) cells in the ADCC/ADCP assay.

### ADCC and ADCP assay

ADCC/ADCP activity of PBMCs was measured in a three-color flow cytometric assay [31,32]. This recently established method is capable of providing data for ADCC and ADCP performance simultaneously. SKBR3 cells (1 × 10^6^) were incubated with 1 μl of 0.5 mM carboxyfluorescein diacetate succinimidyl ester (CFSE; Invitrogen, Eugene, OR, USA) in PBS for 10 min at 37°C. Cells were washed in ice cold SKBR3 medium and returned to normal cell culture conditions 24 hours prior to the assay. The following day effector cells were mixed with target cells at an E/T ratio of 25:1 (12.5 × 10^6^/ml effector cells, 5 × 10^5^/ml target cells; final volume: 400 μl) and incubated with or without antibodies (trastuzumab, rituximab; Roche, Basel, Switzerland) at a concentration of 2.5 μg/ml for 2.5 hours at 37°C in a humidified atmosphere of 5% CO_2_. Subsequently, effector cells were labeled with CD45 PE (Immunotech, Beckman Coulter, Marseille, France), and dead cells with 7-aminoactinomycin D (7-AAD; eBioscience, San Diego, CA, USA) followed by a three-color flow cytometric analysis, carried out on a Gallios™ device (Beckman Coulter). CFSE & 7AAD double positive cells represented tumor target cells killed by ADCC, whereas CFSE & CD45 double positive cells were defined as tumor cells phagocytized by immune cells (ADCP). The amount of ADCC/ADCP activity was compared between cancer patients and healthy volunteers. Moreover, ADCC/ADCP levels of patients were correlated with different PBMC markers and PFS.

### Flow cytometric analysis of PBMCs

For the evaluation of T_reg_ cells, IntraPrep (Immunotech) was used for fixation and permeabilization followed by staining with anti-forkhead-box-protein 3 (FOXP3) PE (eBioscience), anti-CD4 ECD (Immunotech) and anti-CD25 FITC (BD Pharmingen, Franklin Lakes, NJ, USA) antibodies. Natural killer (NK) cells were defined by anti-CD56 FITC (Sigma Aldrich). Monocytes were labeled with anti-CD14 FITC (BD Pharmingen). Expression of FcγRI was achieved by staining with anti-CD64 PC5 (Immunotech), FcγRII with anti-CD32 PE (Acris Antibodies GmbH, Herford, Germany) and FcγRIII with anti-CD16 PE (Immunotech). The lysosomal-associated membrane protein 1 (LAMP-1) expression was determined by anti-CD107a FITC (BD Pharmingen). IgG1 (Mouse) FITC/PE, IgG1 (Mouse) PC5 and IgG1 (Mouse) ECD (Immunotech) antibodies served as isotype controls.

### Statistical analysis

Statistical analyses were based on non-parametric tests and carried out with IBM SPSS Statistics 20.0.0 software (SPSS Inc., Chicago, IL, USA). Differences between the 3 groups were established using the Mann–Whitney-U-Test. Correlations between the measured parameters were assessed using Spearman’s rank correlation coefficient. A receiver operating characteristic curve (ROC) was conducted to estimate a cut-off value for PFS. Kaplan-Meier curves were calculated to show PFS within the period of the follow-up, and were compared with a Log-Rank test. For all statistical tests a two-sided p-value of ≤ 0.05 was considered significant.

Data presented by box- and whiskers-plots: The first and third quartiles are shown as boxes, including the median. The whiskers display 1.5 times the interquartile range (IQR). Dots represent outliers between 1.5 and 3 times the IQR. Triangles symbolize extreme outliers above 3 times the IQR.

### Ethics statement

This study protocol was approved by the “Ethics Committee of the Medical University of Vienna” (#445/2010). All patients and healthy volunteers gave consent after obtaining written information.

## Results

**Figure 1:**
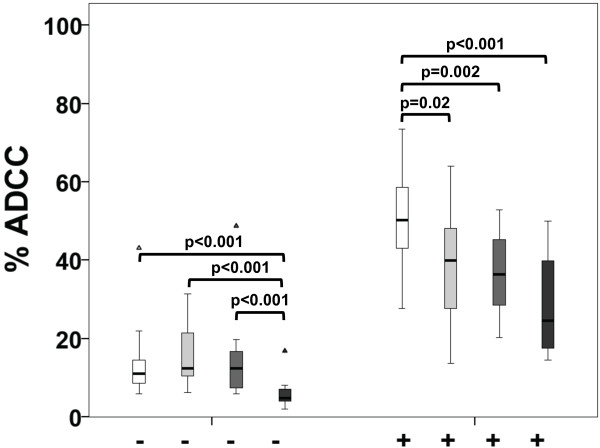
**Trastuzumab-mediated ADCC of healthy volunteers and breast cancer patients.** The extent of ADCC was determined by flow cytometry and is shown in % of total tumor cell counts. An E/T ratio of 25:1 was used. White boxplots represent healthy volunteers, light grey adjuvant, grey metastatic and dark grey trastuzumab naive (t-naive) breast cancer patients. The addition of trastuzumab is indicated at the bottom (-/+).

First, we determined the potential of HER2/neu antibody-specific ADCC and ADCP in breast cancer patients. HER2/neu-specific ADCC was significantly reduced in the adjuvant (p = 0.02), metastatic (p = 0.002) and t-naive group (p < 0.001), when compared to healthy controls (Figure 1). Similar results were obtained with an E/T ratio of 12.5:1 (Additional file 1: Figure S1). For trastuzumab treatment-naive patients we observed lower baseline ADCC activity compared to all other groups (p < 0.001).

**Figure 2:**
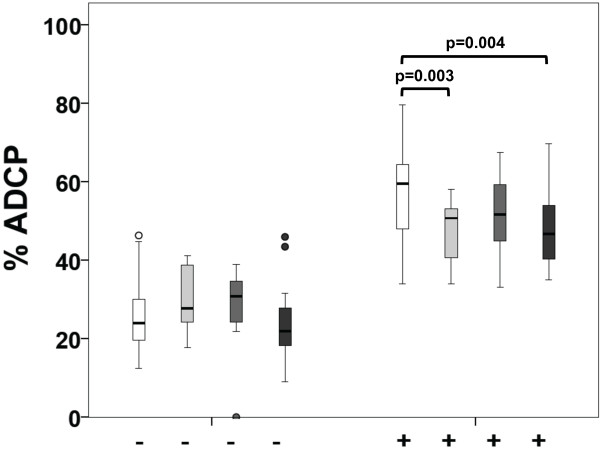
**Trastuzumab-mediated ADCP of healthy volunteers and breast cancer patients.** The extent of ADCP was determined by flow cytometry and is shown in % of total tumor cell counts. An E/T ratio of 25:1 was used. White boxplots represent healthy volunteers, light grey adjuvant, grey metastatic and dark grey trastuzumab naive (t-naive) breast cancer patients. The addition of trastuzumab is indicated at the bottom (-/+).

**Figure 3:**
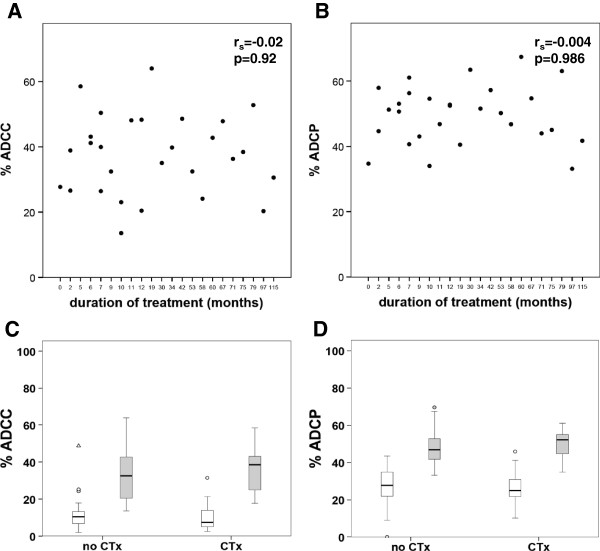
**Influence of treatment duration and chemotherapy applied in the last 3 months on trastuzumab-mediated ADCC/ADCP.** The extent of ADCC/ADCP was determined by flow cytometry and is shown in % of total tumor cell counts. An E/T ratio of 25:1 was used. White boxplots represent baseline ADCC/ADCP without trastuzumab (-) and light grey stimulation with trastuzumab (+). Applied chemotherapy (CTx) is indicated on the bottom. **A, C)** ADCC. **B, D)** ADCP.

Analyzing ADCP, we observed significant reduction in the adjuvant group (p = 0.003) and t-naive group (p = 0.004) compared to healthy controls. In metastatic patients, a tendency towards reduced ADCP was observed (Figure 2). Here it is notable that adjuvant patients were treated with anti-HER2/neu antibodies for a median time of 7 months (IQR = 5), while metastatic patients were treated for 58 months (IQR = 45). However, neither treatment duration nor chemotherapy within the preceding 3 months had an effect on ADCC (Figure 3A, C) or ADCP efficiency (Figure 3B, D). Similar results were obtained using an E/T ratio of 12.5:1 (Additional file 2: Figure S2).

As the main effector cells of antibody-mediated tumor cell killing of PBMCs are monocytes (CD14+) and NK cells (CD56+), we investigated the singular contributions of these two immune cell populations on ADCC and ADCP activity. NK cells triggered in the presence of trastuzumab substantial and significant levels of ADCC (p = 0.002) but not of ADCP, whereas the picture was completely opposite for monocytes (Additional file 3: Figure S3A, B). Monocytes achieved high levels of phagocytosis (p = 0.001) but no significant cytotoxicity.

Concerning complement-dependent cytotoxicity (CDC), a trend towards trastuzumab-mediated CDC of cancer cells was observed (Additional file 4: Figure S4, Additional file 5: Supplementary methods), but this effect was not significant. This is in line with previous studies stating that CDC is indeed induced, but is not a major mechanism of action for trastuzumab, except in combination with CDC-enhancing agents [33].

**Figure 4:**
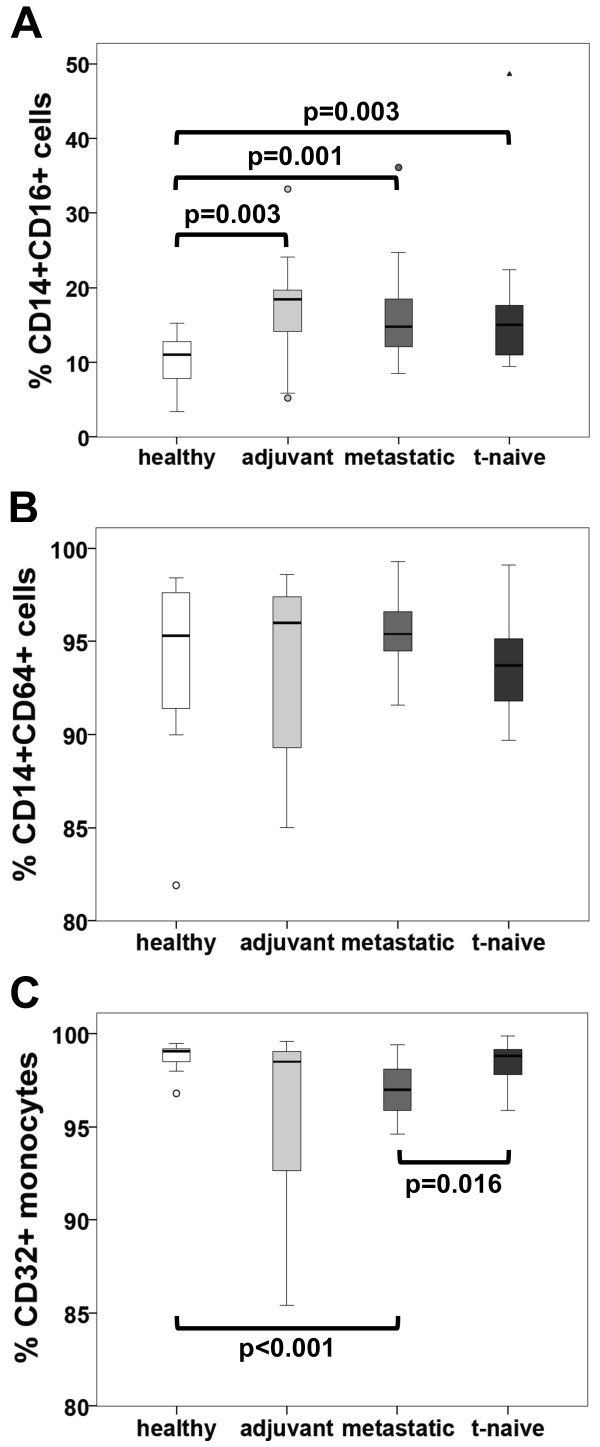
**Frequency of CD16, CD32 and CD64 expression on monocytes of healthy volunteers and breast cancer patients determined by flow cytometry. A)** Frequency of CD14+CD16+ cells of total CD14+ cells is given in %. **B)** Frequency of CD14+C64+ cells of total CD14+ cells is given in %. **C)** The frequency of CD32+ on monocytes (identified using FSC and SSC) is given in %. Patient cohorts are indicated at the bottom.

**Figure 5:**
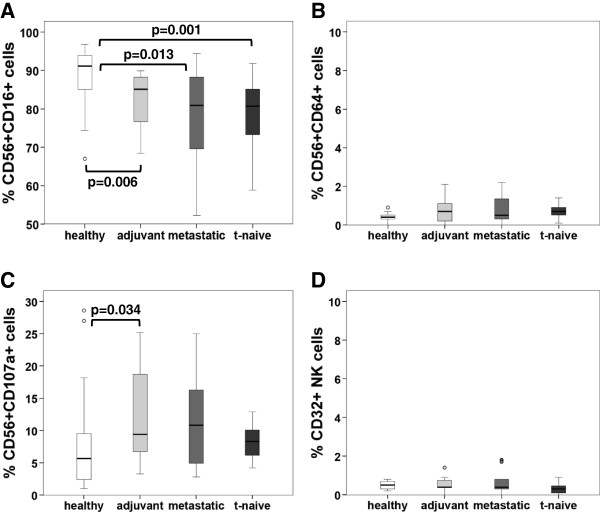
**Frequency of CD16, CD32, CD64 and CD107a expression on NK cells of healthy volunteers and breast cancer patients determined by flow cytometry. A)** The frequency of CD56+CD16+ cells of total CD56+ cells is given in %. **B)** Frequency of CD56+CD64+ cells of total of CD56+ cells is given in %. **C)** Frequency of CD56+C107a+ cells of total CD56+ cells is given in %. **D)** The frequency of CD56+CD32+ of total CD56+ cells using lymphocyte gate (defined by FSC and SSC) is given in %. Patient cohorts are indicated at the bottom.

**Figure 6:**
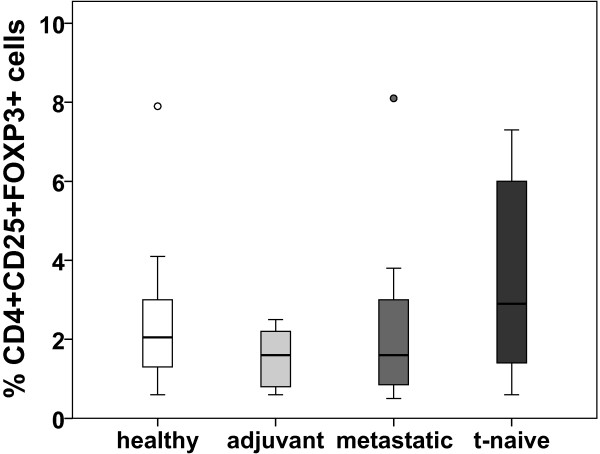
**Frequency of CD4+CD25+FOXP3+ cells (T**_**reg**_**) of total CD4+ cells of healthy volunteers and breast cancer patients.** The frequency of CD25+FOXP3+ cells of total CD4+ cells was determined by flow cytometry and is given in %. Patient cohorts are indicated at the bottom.

ADCC and ADCP are mediated by subpopulations of CD56+ and CD14+ cells, respectively. Therefore, we determined the expression of FcγRI (CD64), FcγRII (CD32) and FcγRIII (CD16) on the above-mentioned subpopulations in our patients. Expression of CD16 was significantly increased in CD14+ cells of patients treated in a metastatic or adjuvant setting as well as in the t-naive group (p = 0.001, p = 0.003 and p = 0.003 respectively), as compared to healthy controls (Figure 4A). In contrast, CD56+ cells showed a decrease of CD16 in the adjuvant (p = 0.006), metastatic (p = 0.013) and t-naive (p = 0.001) group (Figure 5A). The expression of CD32 on monocytes showed differences between the study cohorts (Figure 4C). However, on NK cells this receptor was hardly detectable (Figure 5D). CD64 expression was consistent on CD14+ cells in all cohorts (Figure 4B), but almost undetectable on CD56+ cell (Figure 5B). LAMP-1 (CD107a), a degranulation marker indicating cytotoxic activation [34,35], was up regulated in the adjuvant cohort (p = 0.034), as compared to healthy controls (Figure 5C). The percentage of T_reg_ cells of all CD4+ cells was not significantly altered in any of the groups (Figure 6).

**Figure 7:**
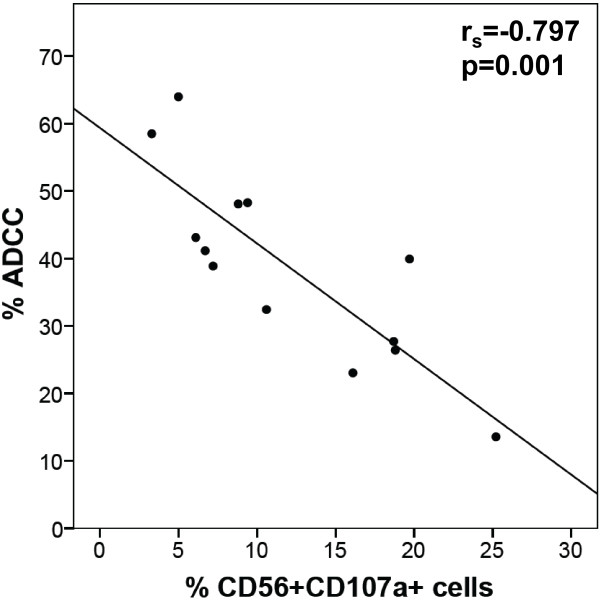
**Correlation of trastuzumab-mediated ADCC with the frequency of CD107a expression on NK cells of adjuvant patients.** The frequency of CD56+CD107a+ cells of total CD56+ cells were correlated with ADCC (E/T ratio 25:1) using Spearman’s rank correlation coefficient.

Moreover, we evaluated whether there was a correlation between HER2/neu-specific ADCC/ADCP activity and any CD14+ or CD56+ subpopulation measured above. Patients in the adjuvant setting showed negative correlations between CD56+CD107a+ cells (Figure 7; r_s_ = -0.797) and ADCC. Similar results were obtained at an E/T ratio of 12.5:1 (Additional file 6: Figure S6; r_s_ = -0.8). In healthy controls, metastatic and t-naive patients there was no correlation between any investigated marker on PBMCs and ADCC/ADCP activity.

**Figure 8:**
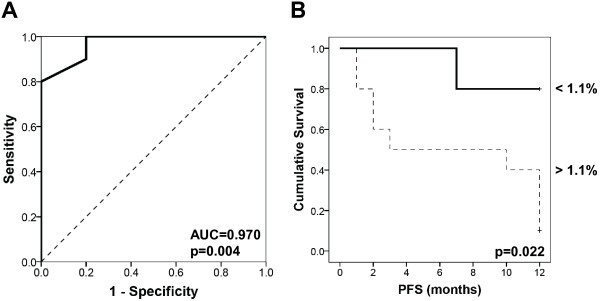
**Correlation between the frequency of T**_**reg **_**cells and PFS in metastatic breast cancer patients. (A)** ROC chart. The area under the curve served for the analysis of potentially diagnostic T_reg_ cells’ sensitivity and specificity to assess the predictive value of T_reg_ cells on PFS. **(B)** Kaplan Meier curves evaluating this possible T_reg_ cut-off value within 12 months of follow-up.

We analyzed the predictive value of the parameters mentioned above for PFS in metastatic patients assessed in a 1-year follow-up. For this purpose, ROC analysis was applied to measure the prognostic power by the area under the curve, and to determine a threshold for prediction of PFS. This revealed that only the number of T_reg_ cells could significantly predict PFS (Figure 8A; AUC = 0.970, p = 0.004). Moreover, taking the cut-off value at 1.1% of T_reg_ cells of the amount of CD4+ cells resulted in a diagnostic sensitivity and specificity of 90% and 80%, respectively. There was a significant difference (p = 0.022) between the groups above and below this value and permitted a hypothetical prediction of PFS depicted by Kaplan-Meier curves (Figure 8B).

## Discussion

In this study, we demonstrated that neither disease progression nor duration of antibody treatment or chemotherapy had an impact on ADCC in HER2/neu breast cancer patients. This finding, that trastuzumab-mediated ADCC levels were not influenced by the treatment duration, is in line with the recent PHARE trial, which affirmed the recommendation for a 12-month standard treatment compared to 6 months of adjuvant treatment [36]. Nonetheless ADCC was lower in breast cancer patients as compared to healthy controls. This is also the first study indicating ADCP occurrence in this type of patients during treatment. In contrast to the popular belief that the immune mechanisms of antibody treatment deteriorate in metastatic cancer [30], our data indicates that trastuzumab stimulates ADCC in the metastatic setting as effectively as in the adjuvant setting. This supports the current clinical concept that trastuzumab treatment can be applied beyond disease progression [37-39] and at the same time underlines the relevance of ADCC as the major mechanism at any stage of the disease.

Chemotherapy associated immunosuppression leading to reduced levels of activated CD56+ cells in breast cancer [40] appears to play a minor role for trastuzumab-dependent ADCC. This could be attributed to the fact that chemotherapy regimens of breast cancer patients frequently contain taxanes, which are known to stimulate the immune system [41].

Analyzing cell types that are relevant for antibody therapy, we found that CD16 expression on CD56+ cells was reduced in all patient cohorts. Previously, it had been shown in an *in vitro* stimulated cell culture trial that ADCC activity leads to a reduction of this cell type [26]. Thus, we hypothesize that one reason for the reduction of these particular cells in patient samples may be that ADCC had already occurred in patients. We favor this hypothesis over the notion that CD56+CD16+ cells are reduced in tumors due to immunosuppression, as we did not see a difference between adjuvant and metastatic patients, the latter being likely more affected by immunosuppression. Since we observed lower ADCC activity in all patients as compared to healthy control subjects, we suggest that treatment might potentially lead to an inhibition of ADCC, in part due to a decrease in ADCP reactivity. Thus, adjuvant immunotherapies need to be developed to counteract this effect. Moreover, we did not see a correlation between CD56+CD16+ cells and ADCC as observed by others [26]. An explanation for this could be the fact, that Varchetta et al. assessed ADCC prior to therapy or early during therapy, whereas we determined immune parameters after prolonged therapy.

We also found an up regulation of CD107a on CD56+ cells in the adjuvant patient cohort. CD107a up regulation has also been shown to occur *in vitro* following ADCC, as this protein is a parameter for degranulation [34,35]. Thus, we again hypothesize that the modulation of this receptor is an indication that ADCC had occurred *in vivo* in those patients rather than representing a therapy-independent up regulation. Thus, increased CD107a expression could be interpreted as a sign for ADCC still occurring in the patient 3 weeks after antibody administration.

Although we found less CD56+ cells expressing CD16 in treated patients, we found a higher frequency of CD16 on CD14+ cells in patients as compared to healthy controls. This showed that ADCP is not associated with a down regulation of cells expressing CD16. An increase of CD14+CD16+ cells might rather be a sign of activation, as this cell type is reported to be increased in patients with acute and chronic infections undergoing hemodialysis [42]. Alternatively, an up regulation of this cell type might be a sign of the malignant disease itself, as intermediate monocytes including CD14+CD16+ cells had been enhanced in tumor patients [43]. This is in line with a study by Feng et al. who even associated an increase of CD14+CD16+ cells with tumor size and pathological staging [44]. Taking the up regulation of CD16 and its affinity to trastuzumab Fc into consideration remains speculative because we did not look at soluble HER2/neu in our patients’ sera. It could be that trastuzumab forms complexes with this soluble receptor and binds more efficiently to patients’ monocytes that have up regulated CD16. But as our assay was done with isolated PBMCs, tumor cells and artificial addition of trastuzumab, no patient serum is included. Therefore we could not address this phenomenon in our experimental approach.

Furthermore, we analyzed the CD64 expression on CD14+ and CD56+ cells, as this receptor has been documented as a direct potent trigger molecule for ADCC and phagocytosis [45,46]. CD64 was constitutively expressed on monocytes and not altered within any of the groups. On CD56+ cells, CD64 expression was almost undetectable as they usually do not express this receptor.

Additionally, we investigated CD32 expression, a receptor which is involved in phagocytosis. Recently, expression of CD32 in metastatic melanoma has been correlated with an impairment of tumor susceptibility to IgG-dependent cellular response [47]. We did not detect any CD32 expression on our SKBR3 cell line (data not shown), confirming the lack of this receptor in other cancers [48]. Usually monocytes consistently express activating CD32, while inhibitory CD32 is only poorly expressed [49,50], which rather speaks for its role as an activating receptor on those cells. In our study we found a high expression of CD32 on monocytes. However, reduced expression levels did not necessarily correlate with reduced phagocytic activity as monocytes of the t-naive group had an almost normal CD32 expression level but reduced phagocytic activity. We did not find CD32 expression on NK cells, which supports the notion that CD32 is primarily expressed on monocytes and is enriched during phagocytosis [51]. Recently, a novel polymorphism affecting the locus encoding human Fc receptors has been reported that leads to inhibitory CD32 expression on NK cells and has been shown to negatively regulate IgG-induced NK cell activation [52]. Obviously, this mechanism does not apply for our patients.

The initial goal of this study was to determine an immune marker that correlates with ADCC or ADCP activity. Our findings showed negative correlations between CD56+CD107a+ cells and ADCC in the adjuvant setting, which could possibly be a sign of immune activation. Thus, effective therapy might be restrained by chronic stimulation of effector cells.

We are aware of the fact that the healthy volunteers enrolled in our study were younger compared to the investigated breast cancer patients. However, we did not find any correlation between age and ADCC/ADCP activity in patients or healthy volunteers. This is in line with studies on CD56+ and CD14+ cells, reporting that the number of both immune populations is preserved or increases with age [53,54] and, although the per-cell activity for cytotoxicity decreases or stays the same, the amount of ADCC is maintained [53]. Therefore, we hypothesize that age-dependent immune-hyporeactivity does not have an impact on our findings.

It is important to note that in this study we used an excess of antibodies in our *ex vivo* ADCC and ADCP assay as had also been done by Varchetta et al. [26]. This gave us the opportunity to focus on the cellular reactivity of the patients’ immune system.

Interestingly, peripheral T_reg_ cells, which had been demonstrated to be up regulated during disease progression [55], were not observed to be significantly higher in metastatic patients compared to healthy volunteers, adjuvant or t-naive patients in our study. Moreover, no correlation between the number of T_reg_ cells and ADCC or ADCP activity could be observed. One reason might be the fact that trastuzumab itself can lead to a reduction of T_reg_ cells [56]. Whether or not this is an effect of chronic immune stimulation by trastuzumab therapy as discussed above remains to be elucidated. Despite a possible down regulation of T_reg_ cells by trastuzumab, they were negatively correlated with disease progression in metastatic cancer patients. This effect could be even more prominent in tumor tissue. This indicates that T_reg_ cells are of biological relevance within the metastatic patient cohort [57-60]. It should be noted that the number of T_reg_ cells in peripheral blood might not be representative of the number of T_reg_ cells infiltrating into the tumor or its surrounding tissue.

We are further aware that ADCC also correlates with FcγR-polymorphisms on CD56+CD16+ lymphocytes [26,61-63], but we did not address this point in the present study. To which extent these polymorphisms affect total immune-cell killing mediated by trastuzumab remains unclear and should be further investigated.

## Conclusion

We conclude from our study that trastuzumab-mediated ADCC is reduced in breast cancer patients compared to healthy volunteers but is not further down regulated by disease progression, chemotherapy or prolonged antibody application. Furthermore, results of our work propose T_reg_ cells as a potential prognostic factor in metastatic breast cancer.

## Abbreviations

ADCC: Antibody-dependent cell-mediated cytotoxicity; ADCP: Antibody-dependent cell-mediated phagocytosis; PMBCs: Peripheral blood mononuclear cells; Treg: Regulatory T cells; FOXP3: Forkhead-box-protein 3; NK: Natural killer; Th2: Type 2 helper T cells; FcγR: Fc-gamma receptor; LAMP1: Lysosomal-associated membrane protein 1; TNF-α: Tumor necrosis factor alpha; IL: Interleukin; HER2/neu: Human epidermal growth factor receptor 2; mAb: Monoclonal antibody; TAA: Tumor-associated antigens; CTx: Chemotherapy; E: Effector; T: Target; RT: Room temperature; CFSE: Carboxyfluorescein diacetate succinimidyl ester; 7-AAD: 7-aminoactinomycin D; FITC: Fluorescein isothiocyanate; PE: Phycoerythrin; APC: Allophycocyanin; ECD: Phycoerythrin-Texas Red conjugate (energy coupled dye); PC5: Phycoerythrin-cyanine 5 conjugate; FCS: Fetal calf serum; DMSO: Dimethyl sulfoxide; EDTA: Ethylene diamine tetra-acetic acid; IQR: Interquartile range; AUC: Area under the curve; ROC: Receiver operating characteristic; PFS: Progression-free survival; RECIST: Response evaluation criteria in solid tumors.

## Competing interests

The authors declare that they have no competing interests.

## Authors’ contributions

BP designed, performed the experiment and wrote the manuscript. JL performed the experiment, carried out the statistical analysis and wrote the manuscript. JS, EJJ, JF and MS designed the experiment. GS and RB enrolled the patients and critically analyzed the data. MB designed the experiment and wrote the manuscript. All authors read and approved the final manuscript.

## Supplementary Material

Additional file 1: Figure S1 Trastuzumab-mediated ADCC of healthy volunteers and breast cancer patients. The extent of ADCC was determined by flow cytometry and is shown in % of total tumor cell counts. An E/T ratio of 12.5:1 was used. White boxplots represent healthy volunteers, light grey adjuvant, grey metastatic and dark grey trastuzumab naive (t-naive) breast cancer patients. The addition of trastuzumab is indicated at the bottom (-/+). Click here for file

Additional file 2: Figure S2 Influence of treatment duration and chemotherapy applied in the last 3 months on trastuzumab-mediated ADCC. The extent of ADCC was determined by flow cytometry and is shown in % of total tumor cell counts. An E/T ratio of 12.5:1 was used. White boxplots represent baseline ADCC without trastuzumab (-) and light grey stimulation with trastuzumab (+). Applied chemotherapy (CTx) is indicated on the bottom. Click here for file

Additional file 3: Figure S3 Trastuzumab-mediated ADCC/ADCP of purified NK cells and monocytes of healthy volunteers. The extent of ADCC/ADCP was determined by flow cytometry and is shown in % of total tumor cell counts. An E/T ratio of 25:1 was used. White boxplots represent baseline ADCC/ADCP without trastuzumab (-) and light grey stimulation with trastuzumab (+). Different cell populations are indicated at the bottom. A) ADCC. B) ADCP. Click here for file

Additional file 4: Figure S4 Trastuzumab-mediated CDC of healthy volunteers. The extent of CDC was determined by flow cytometry and is shown in % of total tumor cell counts. An amount of 25% human serum was used. The addition of trastuzumab is indicated at the bottom (-/+). Click here for file

Additional file 5 Supplementary methods. Click here for file

Additional file 6: Figure S6 Correlation of trastuzumab-mediated ADCC with the frequency of CD107a expression on NK cells of adjuvant patients. The frequency of CD56+CD107a+ cells of total CD56+ cells were correlated with ADCC (E/T ratio 12.5:1) using Spearman’s rank correlation coefficient. Click here for file
