# Supplementary figures and images for: Trastuzumab mediates antibody-dependent cell-mediated cytotoxicity and phagocytosis to the same extent in both adjuvant and metastatic HER2/neu breast cancer patients
Source: J Transl Med. 2013 Dec 12;11:307. doi: 10.1186/1479-5876-11-307 (PMC4029549; doi:10.1186/1479-5876-11-307)

**% ADCC**

100  
80  
60  
40  
20  
0

-

-

-

-

+

+

+

+

$p < 0.001$

$p < 0.001$

$p < 0.001$

$p = 0.002$

$p < 0.001$

$p < 0.001$

0

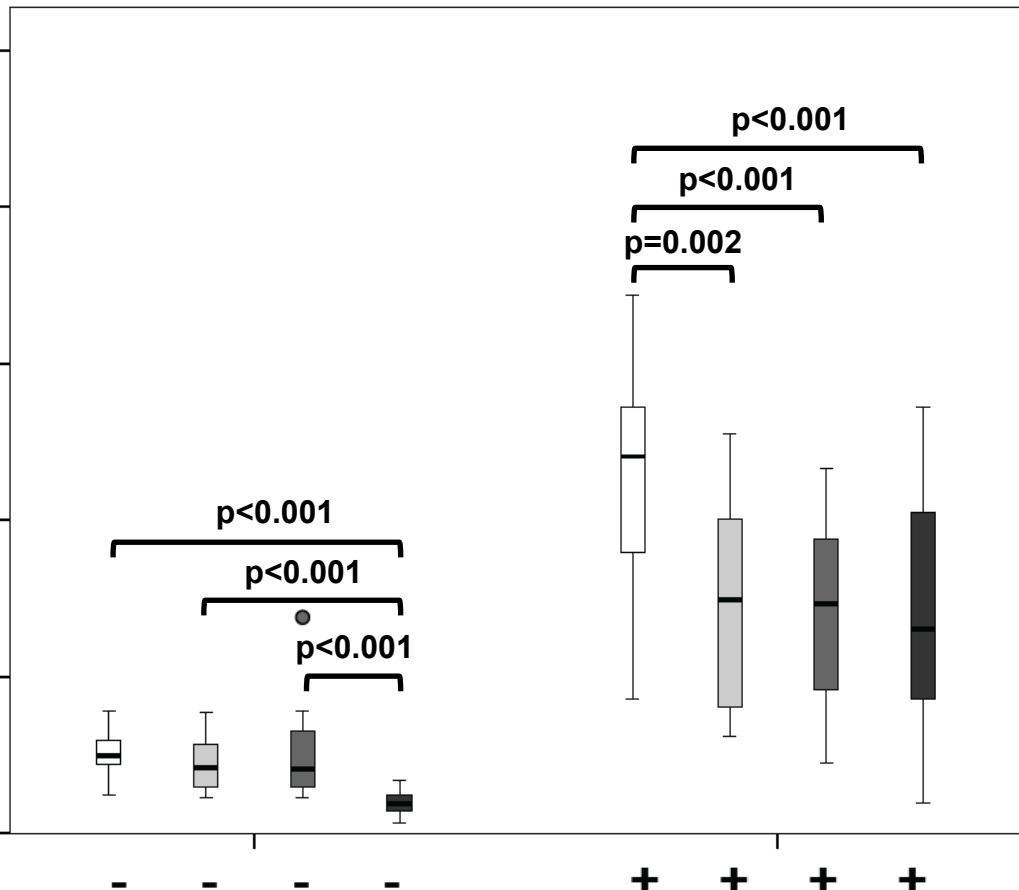

Supplement: Additional file 1: Figure S1 — Trastuzumab-mediated ADCC of healthy volunteers and breast cancer patients. The extent of ADCC was determined by flow cytometry and is shown in % of total tumor cell counts. An E/T ratio of 12.5:1 was used. White boxplots represent healthy volunteers, light grey adjuvant, grey metastatic and dark grey trastuzumab naive (t-naive) breast cancer patients. The addition of trastuzumab is indicated at the bottom (-/+). [file 1479-5876-11-307-S1.pdf]

**A**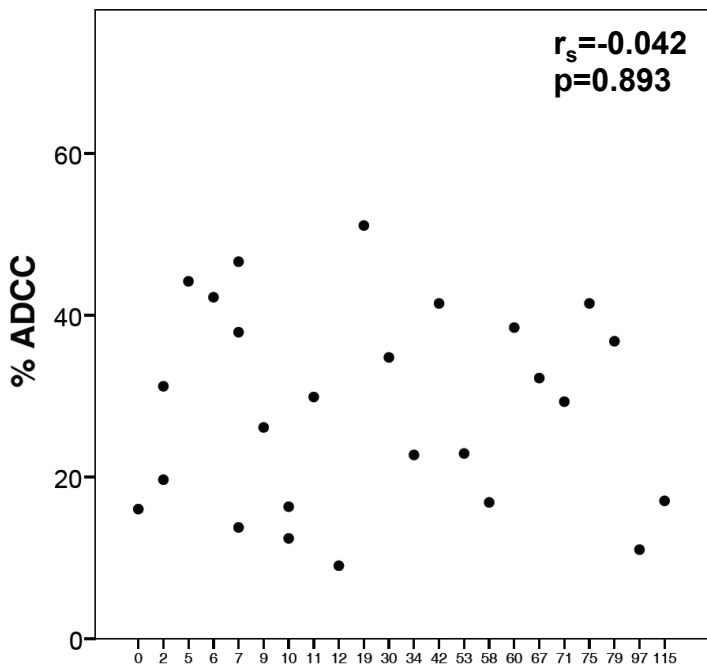**B**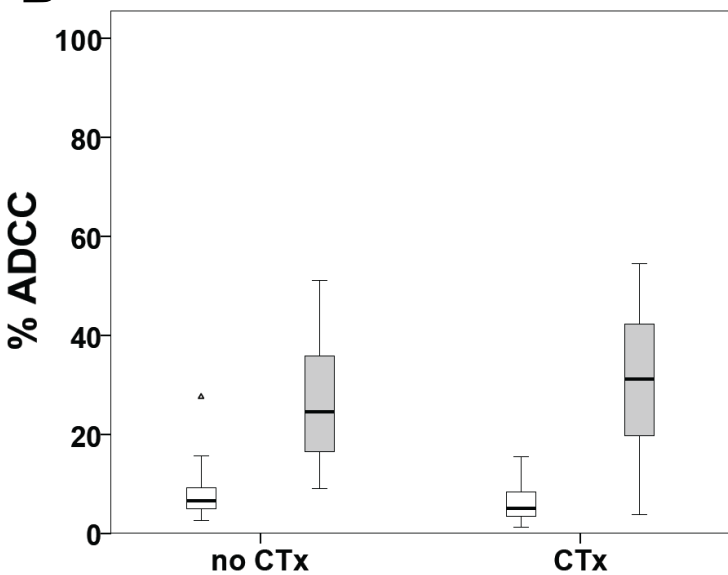

Supplement: Additional file 2: Figure S2 — Influence of treatment duration and chemotherapy applied in the last 3 months on trastuzumab-mediated ADCC. The extent of ADCC was determined by flow cytometry and is shown in % of total tumor cell counts. An E/T ratio of 12.5:1 was used. White boxplots represent baseline ADCC without trastuzumab (-) and light grey stimulation with trastuzumab (+). Applied chemotherapy (CTx) is indicated on the bottom. [file 1479-5876-11-307-S2.pdf]

**A**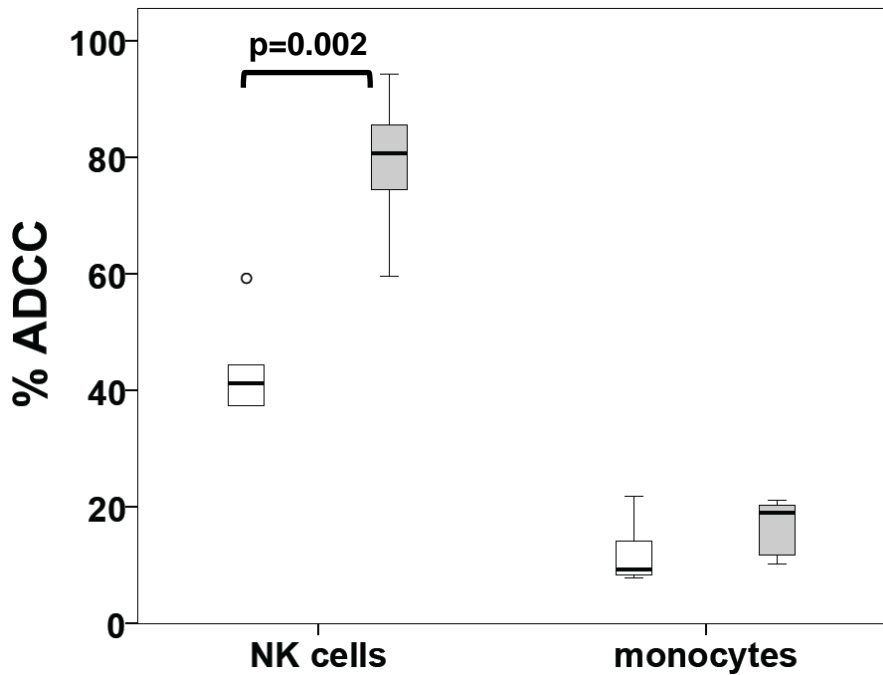**B**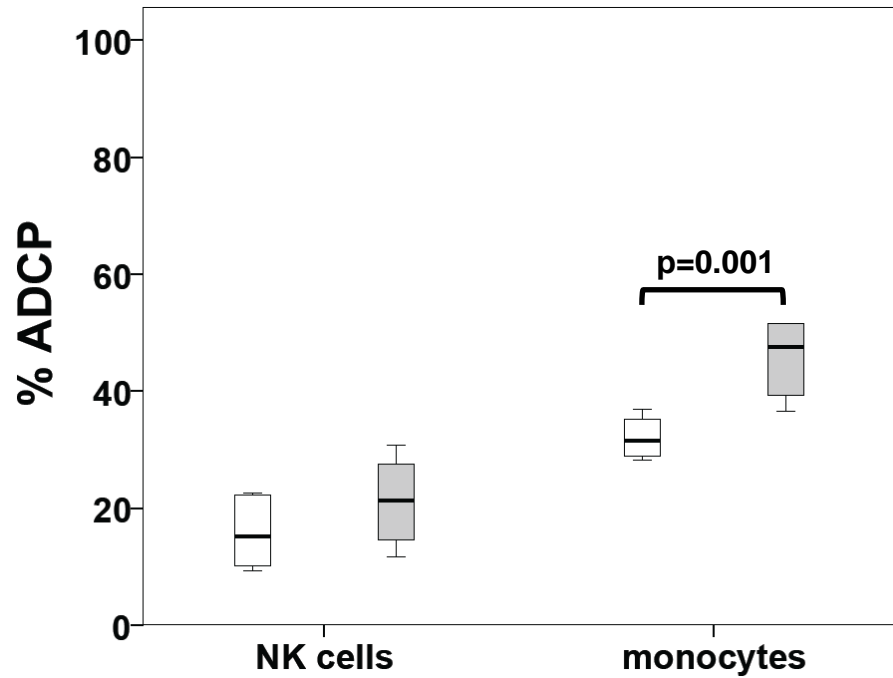

Supplement: Additional file 3: Figure S3 — Trastuzumab-mediated ADCC/ADCP of purified NK cells and monocytes of healthy volunteers. The extent of ADCC/ADCP was determined by flow cytometry and is shown in % of total tumor cell counts. An E/T ratio of 25:1 was used. White boxplots represent baseline ADCC/ADCP without trastuzumab (-) and light grey stimulation with trastuzumab (+). Different cell populations are indicated at the bottom. A) ADCC. B) ADCP. [file 1479-5876-11-307-S3.pdf]

**% CDC**

**100**  
**80**  
**60**  
**40**  
**20**  
**0**

**-**

**+**

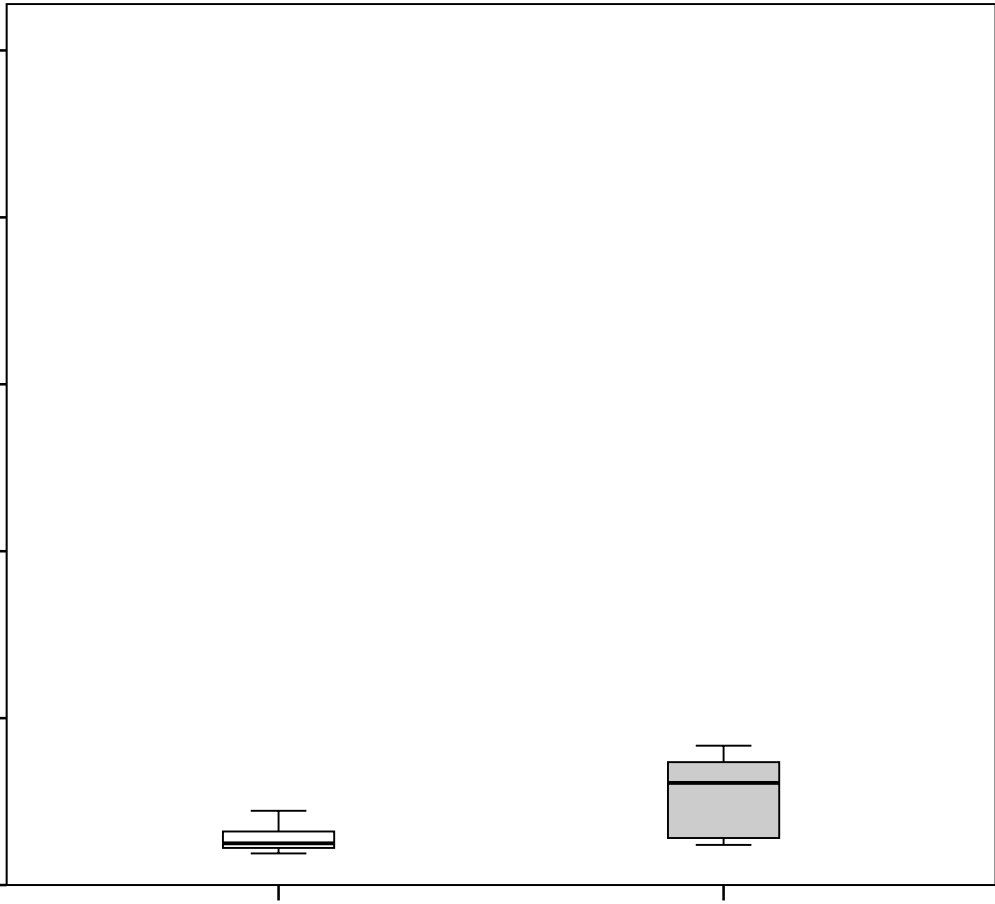

Supplement: Additional file 4: Figure S4 — Trastuzumab-mediated CDC of healthy volunteers. The extent of CDC was determined by flow cytometry and is shown in % of total tumor cell counts. An amount of 25% human serum was used. The addition of trastuzumab is indicated at the bottom (-/+). [file 1479-5876-11-307-S4.pdf]

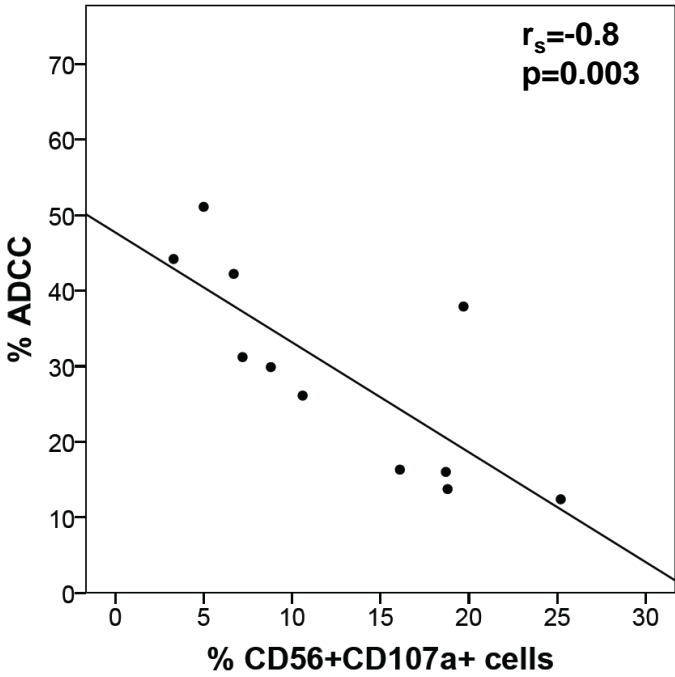

Supplement: Additional file 6: Figure S6 — Correlation of trastuzumab-mediated ADCC with the frequency of CD107a expression on NK cells of adjuvant patients. The frequency of CD56+CD107a+ cells of total CD56+ cells were correlated with ADCC (E/T ratio 12.5:1) using Spearman’s rank correlation coefficient. [file 1479-5876-11-307-S6.pdf]
