# Supplementary material for: Trastuzumab mediates antibody-dependent cell-mediated cytotoxicity and phagocytosis to the same extent in both adjuvant and metastatic HER2/neu breast cancer patients
Source: J Transl Med. 2013 Dec 12;11:307. doi: 10.1186/1479-5876-11-307 (PMC4029549; doi:10.1186/1479-5876-11-307)
Supplement: Additional file 5 — Supplementary methods. [file 1479-5876-11-307-S5.doc]

**Supplementary methods**

**ADCC and ADCP assay with purified NK cells and monocytes:** ADCC and ADCP activity of monocytes and NK cells of 3 healthy volunteers were measured in the three-color flow cytometric assay analogous to the assay with PBMCs. Only prior to Ficoll-Paque separation, whole blood was incubated for 10 min at room temperature with either a monocyte or NK cell enrichment cocktail (STEMCELL Technologies SARL, Grenoble, France). Afterwards effector cells were mixed with target cells at an E/T ratio of 25:1. NK cells were labeled with anti-CD45 PE (Immunotech) and monocytes with anti-CD89 PE (BD Pharmingen). Cells, which underwent cytotoxic killing, were stained with 7-AAD (eBioscience). Analysis was carried out on a dual laser FACSCalibur™ (BD Biosciences, Franklin Lakes, New Jersey, USA).

**CDC assay:** Complement dependent cytotoxicity (CDC) of 3 healthy volunteers was measured in a flow cytometric assay. SKBR3 cells (4 × 10^4^) were labeled with CFSE and incubated in SKBR3 medium (300 µl) plus 25% human serum (100 µl) of healthy volunteers (final volume: 400 µl). After 2.5 hours of incubation at 37°C in a humidified atmosphere of 5% CO_2_ in the presence or absence of 2.5 µg/ml trastuzumab, cells were stained with 7-AAD and analysis was performed on a dual laser FACSCalibur™.
